# Is it wise not to include hair and shoe covers in personal protective equipment (PPE) recommendations?

**DOI:** 10.1017/ice.2020.1306

**Published:** 2020-10-29

**Authors:** Chenyu Sun, Mubashir Ayaz Ahmed, Ce Cheng

**Affiliations:** 1 AMITA Health Saint Joseph Hospital Chicago, Chicago, Illinois; 2 The University of Arizona College of Medicine at South Campus, Tucson Arizona

*To the Editor*—Coronavirus disease 2019 (COVID-19), caused by severe acute respiratory syndrome coronavirus 2 (SARS-CoV-2), has been spreading globally for more than half year.^1^ Healthcare workers (HCWs) on COVID-19 floors and units are aware of the higher risk of contracting SARS-CoV-2.^1^ “Routine care can be resumed only with sufficient and adequate personal protective equipment (PPE)” to protect HCWs to ensure continuous patient care during this pandemic.^2^ In China, 4% of confirmed cases in the first month of COVID-19 outbreak occurred among HCWs, with even higher rates in Europe due to delayed recognition of COVID-19 rather than PPE failures.^3^ However, the items included in PPE protocol and policies vary from institution to institution. The US Centers for Disease Control and Prevention (CDC) does not include hair covers and shoe covers in their PPE recommendations for HCWs.^4^

Despite the CDC not including them, hair covers and shoe covers, along with face masks, gowns, gloves, and other PPE are often used to prevent contamination from patient contact and droplets.^1^ A recent study suggested that the shoes of HCWs might serve as a vector of SARS-CoV-2, transferring it from floors in COVID-19 rooms to floors throughout the unit.^5^ This is not surprising because SARS-CoV-2 contamination was common on floors in COVID-19 patient rooms.^5,6^ Although data on how long SARS-CoV-2 can survive on hairs, or whether it is common to have the contamination on hairs of HCWs are very limited, the virus remains viable for hours to days on different materials.^7^ Therefore, the potential contamination on hairs of HCWs may represent risks of nosocomial infection among non–COVID-19 patients.

We believe it is better to be cautious rather than regretful, and HCWs should be provided shoe covers and hair covers as part of PPE when providing care for COVID-19 patients. More studies will also be needed to assess the risk of contamination on human hairs as well as the efficacy of hair and shoe covers in healthcare settings.
